# Adolescent predisposition to binge drinking is associated with differences in inhibitory control MEG event-related fields

**DOI:** 10.3389/fpsyt.2025.1696748

**Published:** 2026-01-13

**Authors:** Luis Fernando Antón-Toro, Danylyna Shpakivska-Bilan, Lucía López-Abad, Alberto Del Cerro-León, Marcos Uceta, Ricardo Bruña, Luis M. García-Moreno, Fernando Maestú

**Affiliations:** 1 Center of Cognitive and Computational Neuroscience, Universidad Complutense de Madrid (UCM), Madrid, Spain; 2 Department of Experimental Psychology, Cognitive Processes and Speech Therapy, Universidad Complutense de Madrid (UCM), Madrid, Spain; 3 Department of Cellular Biology, Faculty of Biology, Complutense University of Madrid (UCM), Madrid, Spain; 4 Department of Radiology, Complutense University of Madrid, Madrid, Spain; 5 Department of Psychobiology and Methodology in Behavioral Science, Faculty of Education, Complutense University of Madrid (UCM), Madrid, Spain

**Keywords:** adolescence, binge drinking, event related fields, inhibitory control, magnetoencephalography

## Abstract

**Introduction:**

Adolescent binge drinking (BD) is a major public health concern, yet little is known about the neural markers that precede alcohol initiation. This longitudinal study examined whether magnetoencephalography (MEG) event-related fields (ERFs) recorded during inhibitory control predict later BD.

**Methods:**

Eighty-one alcoholnaïve adolescents initially completed a Go/No-Go task during MEG acquisition, alongside self-report measures of impulsivity, sensation seeking, and executive functioning. After two years, 44 participants remained eligible and were classified as controls (CN, n = 20; 10 females) or binge drinkers (BD, n = 24; 12 females) based on reported habits of alcohol consumption.

**Results:**

Behavioral analyses showed no group differences in accuracy in the inhibition task, impulsivity (BIS-11), or executive functioning (BRIEF-SR). The BD group reported higher sensation seeking (SSS-V). When studying electrophysiological activity, cluster-based permutation analyses revealed significant group differences in both the M200 (180–260ms) and M300 (310–510ms) components. In both cases, BD adolescents exhibited larger amplitudes, with sensors localized to left medial and dorsolateral prefrontal areas. These differences were moderate predictors in logistic regression models. Association between ERF and future alcohol use were not influenced by biological sex.

**Discussion:**

The results converge with prior evidence of left prefrontal hyperactivation in adolescent BD and developmental studies showing enhanced recruitment of control networks during adolescence. Findings support the hypothesis that atypical prefrontal executive engagement may represent a vulnerability profile that precedes alcohol use and may contribute to the emergence of BD.

## 1 Introduction

Adolescent binge drinking (BD) is a public health concern due to its high prevalence and adverse consequences (1). It is commonly defined as consuming five or more drinks for males and four or more for females in an approximate timespan of two hours (2), although thresholds and time frames vary across studies (3). The prevalence of BD increases from the age of 14, and acute alcohol intoxication is especially frequent between ages 15 and 24 (4). According to the 2023 NSDUH (5), approximately 33.1% of individuals aged 12–20 have consumed alcohol at least once in their lifetime, while 3.9% of youth aged 12–17 reported binge drinking in the past month. The WHO Regional Office for Europe (6) reports that 57% of 15-year-olds have ever tried alcohol (with slightly higher rates among girls); nonetheless, repeated drunkenness remains common at around 23%. Short-term consequences include a higher risk of acute events such as alcohol intoxication, loss of consciousness, and impulsive behaviors (2), whereas long-term use is associated with disruptions in emotional, social, and behavioral development that interfere with neurobiological processes critical to adolescence (7, 8).

Adolescence is marked by ongoing brain maturation, with the reward-sensitive socioemotional system developing earlier than the prefrontal cognitive control system. This imbalance heightens vulnerability to risk-taking behaviors, including substance use, and affects executive functions such as planning, working memory, flexibility, and inhibition (9–12).

Inhibitory control, the ability to suppress automatic or inappropriate responses to achieve goals (13), depends on a frontoparietal network that includes the right inferior frontal gyrus, supplementary motor area, and primary motor cortex (14, 15). During adolescence, this function continues to specialize through reorganization of these networks (16). The Go/No-Go task assesses inhibitory control by requiring rapid responses to frequent stimuli (Go) and withholding responses to infrequent stimuli (No-Go), thereby engaging top-down control (14). This function can be examined temporally using event-related potentials and fields (ERPs/ERFs), which reflect neural activity phase-locked to external stimuli (17). Several EEG studies on inhibitory control have extensively characterized two key electrophysiological components in ERPs: the N200 (≈200–300ms; M200 in ERFs), linked to conflict monitoring and the initiation of inhibitory processes, and the P300 (≈300–500ms; M300 in ERFs), associated with attentional allocation, cognitive updating, and control. The P300 can be further subdivided into the P3a (orienting) and the P3b (stimulus evaluation and memory updating) (18–20). Magnetoencephalography (MEG), by contrast, measures magnetic fields generated by primary cortical currents, detecting the inflow and outflow of magnetic flux at the scalp. Although ERFs do not present electrical polarity and thus cannot be strictly described as negative or positive components (i.e., N200 or P300), MEG provides millisecond-level temporal resolution with higher spatial accuracy than EEG (17). Importantly, the temporal dynamics of inhibitory processes are preserved across both EEG and MEG, supporting the validity of defining analogous time windows of interest in both modalities.

Among university adolescents and young adults with established BD patterns, inhibitory control alterations have been documented at behavioral and neural levels. Behaviorally, BD youth commit more commission errors and show reduced post-error adjustment (21, 22). Functionally, Go/No-Go and Stop-Signal tasks reveal BOLD hyperactivation in control-related regions, such as the dorsolateral prefrontal cortex, anterior cingulate cortex, and inferior frontal gyrus, consistent with compensatory recruitment to sustain performance (23, 24). At the electrophysiological level, ERP studies similarly report larger No-Go P300 amplitudes and increased right inferior frontal gyrus activation in young BD users, suggesting compensatory mechanisms in the context of less efficient control (25–28). Evidence regarding the N200 is more limited, although reduced No-Go amplitudes—indicative of impaired conflict monitoring—have been described, with similar patterns in adolescent tobacco users (29, 30). Beyond consumption effects, deficits in inhibitory control have been proposed as early markers of vulnerability to BD (31). MEG evidence shows that adolescents who had not yet initiated alcohol use but later developed BD exhibited atypical beta-band hyperconnectivity in frontoparietal networks (dorsolateral prefrontal cortex, right inferior frontal gyrus, posterior parietal cortex) (32, 38). Converging structural and functional findings—such as reduced dorsolateral prefrontal surface area, thinner inferior frontal gyrus, and greater frontal activation during inhibition, also predict subsequent BD and higher impulsivity (33, 34). Likewise, adolescents with a family history of alcohol use disorder (AUD) show ERP alterations, including reduced No-Go P300, prolonged N200 latencies, and lower activation in executive control regions, highlighting premorbid neural risk factors (35–37).

Nevertheless, longitudinal evidence remains scarce in the general adolescent population without a family history of AUD, leaving it unclear whether pre-consumption inhibitory control ERPs/ERFs predict later BD. To address this gap, the present study employed a two-year longitudinal design, recording pre-consumption MEG activity during a Go/No-Go task and focusing on the M200 and M300 components dynamics in relation to drinking patterns two years later. Based on prior work, we hypothesized that (H1) behavioral task measures would not significantly predict future drinking; (H2) lower baseline No-Go M200 amplitude, associated with conflict detection, would be associated with greater subsequent BD, and (H3) higher baseline No-Go M300 amplitude, reflecting increased attentional and executive demands, would predict greater BD levels at follow-up. Confirmation of these hypotheses would support the identification of pre-consumption functional biomarkers useful for early risk detection and for guiding preventive interventions.

## 2 Methods

### 2.1 Participants

Adolescents were recruited from several high schools in the Madrid metropolitan area and evaluated at two time points separated by a two-year follow-up. At baseline, all participants reported no prior alcohol consumption, no family history of alcohol use disorders, and no psychiatric or neurological conditions. To further ensure the absence of early substance use, they completed the Alcohol Use Disorder Identification Test (AUDIT) and a semi-structured interview on drug use habits. During the first evaluation (before the onset of alcohol use), 81 adolescents participated in a magnetoencephalography (MEG) session involving a Go/No-Go inhibitory control task. Additionally, every participant fulfilled psychological and behavioral self-informed scales to measure traits of impulsivity (BIS-11), sensation seeking (SSS-V), and dysexecutive performance (BRIEF-SR). Two years later, 59 of them were reassessed with the AUDIT and interviewed again about their drinking habits. Based on these data, we calculated the number of standard alcohol units (SAUs) typically consumed during a regular 2–3-hour drinking episode, considering both the number and type of beverages. Participants reporting regular tobacco or cannabis use were excluded. We conducted several quality control checks and excluded 15 participants due to the following reasons: substance use (2 participants), poor MEG signal quality: fixed dental retainers (3 participants), excessive movement during the MEG recording (2 participants), excessive muscle artifacts leading to low trial counts after cleaning (3 participants), and low-quality event-related field (ERF) data (i.e., non-identifiable M100 visual component, noisy baseline; 5 participants). After these quality control procedures, the final sample consisted of 44 adolescents, who were subsequently classified into two groups: Low/No drinkers (CN group; SAUs < 3; N = 20; mean age = 14.22 ± 0.42; 10 females) and High/Binge drinkers (BD group; SAUs > 4; N = 24; mean age = 14.31 ± 0.41; 12 females). Written informed consent was obtained from all participants and their parents or legal guardians, in line with the Declaration of Helsinki. The study was approved by the Research Ethics Committee of the Universidad Complutense de Madrid.

### 2.2 Inhibitory control task (go/no-go)

**Figure 1:**
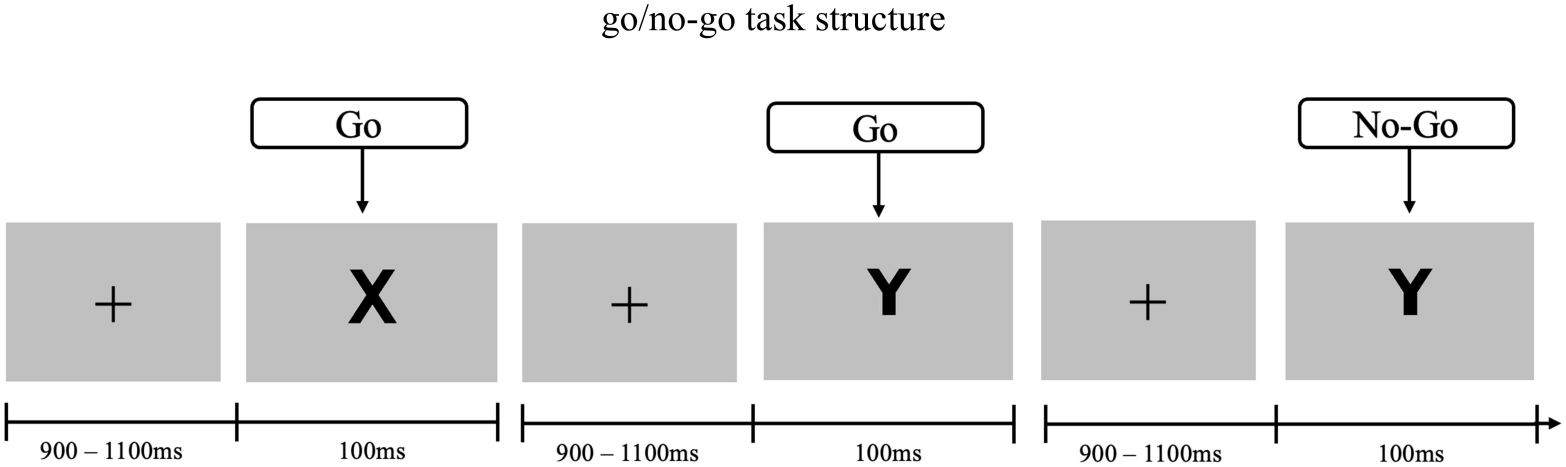
Go/no-go task time-chart and structure. Each trial began with a fixation cross followed by a letter (“X” or “Y”). Go trials required a speeded response to alternating letters, whereas No-Go trials required withholding the response to repeated letters.

Inhibitory tasks are experimental paradigms that assess the ability to suppress automatic or prepotent responses (13). In this study, we employed the Go/No-Go task, a reliable and sensitive measure of motor inhibition. Participants were instructed to fixate on a central cross while visual stimuli (“X” or “Y”) were presented for 100ms, with an interstimulus interval randomly varying between 900 and 1100ms. Go trials (75%) consisted of alternating letters (“X-Y” or “Y-X”), requiring a button press, whereas No-Go trials (25%) involved stimulus repetition (“X-X” or “Y-Y”), requiring response inhibition. **Figure 1** illustrates the temporal sequence of stimuli in the task. The asymmetric distribution of trials was designed to elicit a prepotent response tendency, thereby increasing cognitive demands and enabling a more precise evaluation of inhibitory control. In total, participants completed four blocks of 250 trials each (1000 trials overall). Response hand was counterbalanced across blocks to control lateralization and motor fatigue effects.

### 2.3 Behavioral scales

The BIS-11 questionnaire is a widely used self-report questionnaire that measures trait impulsivity across attentional, motor, and non-planning dimensions. It consists of 30 items rated on a 4-point Likert scale, with higher scores indicating greater impulsivity (39). The BRIEF-SR assesses everyday executive functioning in adolescents through self-report. It includes multiple scales covering inhibition, shifting, emotional control, working memory, and planning/organization, providing a global index of dysexecutive problems (40). The SSS-V evaluates individual differences in the tendency to seek novel, complex, and intense experiences, as well as willingness to take risks for such experiences. It comprises four subscales (thrill and adventure seeking, experience seeking, disinhibition, and boredom susceptibility) (41).

### 2.4 MEG recordings and signal processing

MEG recordings were obtained with a 306-channel Elekta Neuromag system housed in a magnetically shielded room at the Center for Biomedical Technology in Madrid, Spain. Data were sampled at 1,000 Hz with an online anti-alias filter applied in the 0.1–330 Hz range. To reduce environmental noise and compensate for head movements, we applied the temporal extension of the signal space separation method (tSSS; correlation window = 10 s, correlation limit = 0.9) (42). Artifact detection was performed in MATLAB R2020b using the FieldTrip toolbox (43), and all automatically flagged segments were visually inspected by an MEG expert. Epochs were extracted from −300ms to 700ms relative to stimulus onset, using the pre-stimulus interval as baseline. Artifact-free epochs were then segmented into 1,000-sample windows (300 samples baseline + 700 post-stimulus) with an additional 2,000 samples of real data padding on each side.

### 2.5 Event related fields calculation

Event-related fields (ERFs) were computed using the FieldTrip toolbox (43). We selected trials corresponding to correct No-Go responses (successful inhibitions), requiring a minimum of 50 artifact-free trials per participant for inclusion. ERFs were calculated at the sensor level, separately for magnetometers and planar gradiometers, although only magnetometer data were retained for the main analyses. Signals were low-pass filtered at 30 Hz with a FIR filter (order 2000, Hann window), and high-pass filtered at 0.5Hz with an IIR filter (order 2, Butterworth) linearly detrended to remove slow drifts and finally baseline-corrected using the −300ms pre-stimulus interval. Visual inspection of ERFs was carried out across multiple sensor sites (e.g., prefrontal, parietal, and occipital). Following this quality control, 15 participants were excluded due to poor ERF quality (e.g., baseline shifts, absence of the M100 visual component, or excessive high-frequency noise).

### 2.6 Statistical analysis

Group differences in demographic and behavioral measures were examined prior to the electrophysiological analyses. Sex distribution was compared using a chi-square test with Fisher’s exact correction, and age differences were assessed with an independent samples t-test. For the behavioral scales (BIS-11, BRIEF-SR, SSS-V), we performed a multivariate ANCOVA analysis, controlling for sex and age as covariates. All sub-scales were tested individually, and global scales were tested using the sub-scales aggregation as total scores.

For the ERFs analysis, group differences were assessed using a cluster-based permutation test (CBPT) with ANCOVA as the statistical model, including age and biological sex as covariates. Two separate CBPT analyses were performed in predefined time windows: an early window (50–300ms) and a later window (250–600ms), corresponding to the M200 (N200) and M300 (P300) components of inhibitory control (18, 19). This procedure identifies clusters of sensors and time points showing significant differences between consumption groups within these intervals. Clusters were formed from sensors with uncorrected p-values < 0.005, requiring a minimum of three neighboring sensors (≥1% of the array). Cluster-level statistics were derived from a null distribution generated through 10,000 random permutations of the data. Clusters with a permutation-based p-value < 0.05 were considered significant.

Additionally, we conducted two *post-hoc* analysis with the ERF activity obtained in our CBPT analyses. First, we conducted a logistic regression model in order to analyze the predictability of future alcohol consumption groups using ERF activity. Second, we performed a moderation analysis to explore if biological sex influences the association between ERF activity and alcohol use years later.

## 3 Results

### 3.1 Demographic and behavioral scales results

The BD and CN groups did not differ in sex distribution (χ² (1, N = 44) = 0.00, *p* = 1.00; Fisher’s exact *p* = 1.00) or age (t (42) = –0.03, *p* = .98). Regarding behavioral scales and subscales, we conducted ANCOVAs including sex and age as covariates to control for their potential influence. Assumption checks indicated that homogeneity and normality (model residuals) of variances were generally met, with some mild departures. Given that general linear models are robust to moderate violations of these assumptions under balanced samples (44), parametric analyses were retained. Results revealed a significant group effect only on the SSS-V Total score (F = 4.40, *p* = .042), with a medium effect size (η² = .10), indicating higher sensation-seeking levels in the BD group. **Table 1** shows the complete set of results for all subscales.

**Table 1:** ANCOVA analysis of behavioral/cognitive scales.

Measure	Control group		Binge group		F	η²
	M	SD	M	SD		
SSS-V EMO	5.25	3.09	6.54	2.65	2.29	.05
SSS-V EXP	5.10	1.61	5.71	1.48	1.54	.04
SSS-V DES	2.85	1.81	3.87	1.67	3.36	.08
SSS-V BOR	3.05	1.84	3.42	1.38	.46	.01
SSS-V TOT	16.25	5.57	19.54	4.40	4.40*	.10
BRIEF-SR BRI	54.95	8.52	57.46	9.96	.71	.01
BRIEF-SR MCI	63.00	9.99	63.00	10.80	.00	.00
BRIEF-SR GEI	117.95	16.34	120.46	19.62	.21	.00
BIS-11 CIM	16.90	3.94	15.46	4.67	.95	.02
BIS-11 MIM	12.30	6.44	14.58	4.43	2.17	.05
BIS-11 NPIM	19.65	4.15	19.71	7.69	.01	.00
BIS-11 TOT	48.85	9.05	49.71	13.98	.13	.00

Results of Multivariate ANCOVA analysis of behavioral scales and subscales (SSS-V, BRIEF-SR and BIS-11). BD group showed higher sensation seeking scores compared with CN group (bold value). SSS-V, subscales; EMO, Emotions seeking; EXP, Novel experiences seeking; DES, Disinhibition; BOR, Boredom susceptibility; TOT, Total sensation seeking score. BRIEF-SR subscales: BRI, Behavioral regulation index; MCI, Metacognitive index; GEI, Global executive index; BIS-11 subscales: CIM, Cognitive impulsivity; MIM, Motor impulsivity; NPIM, non-planned impulsivity; TOT, Total impulsivity score. *p < 0.05.

### 3.2 Event related potentials and behavioral results

First, we tested behavioral differences in the performance of inhibitory trials (No-Go trials). Shapiro–Wilk tests indicated that No-Go accuracy was normally distributed in both groups (BD: W = 0.97, p = .61; CN: W = 0.94, *p* = .28). An independent samples t-test showed no significant difference between groups (t (42) = 0.18, *p* = .855, 95% CI [–7.12, 8.55]).

**Figure 2:**
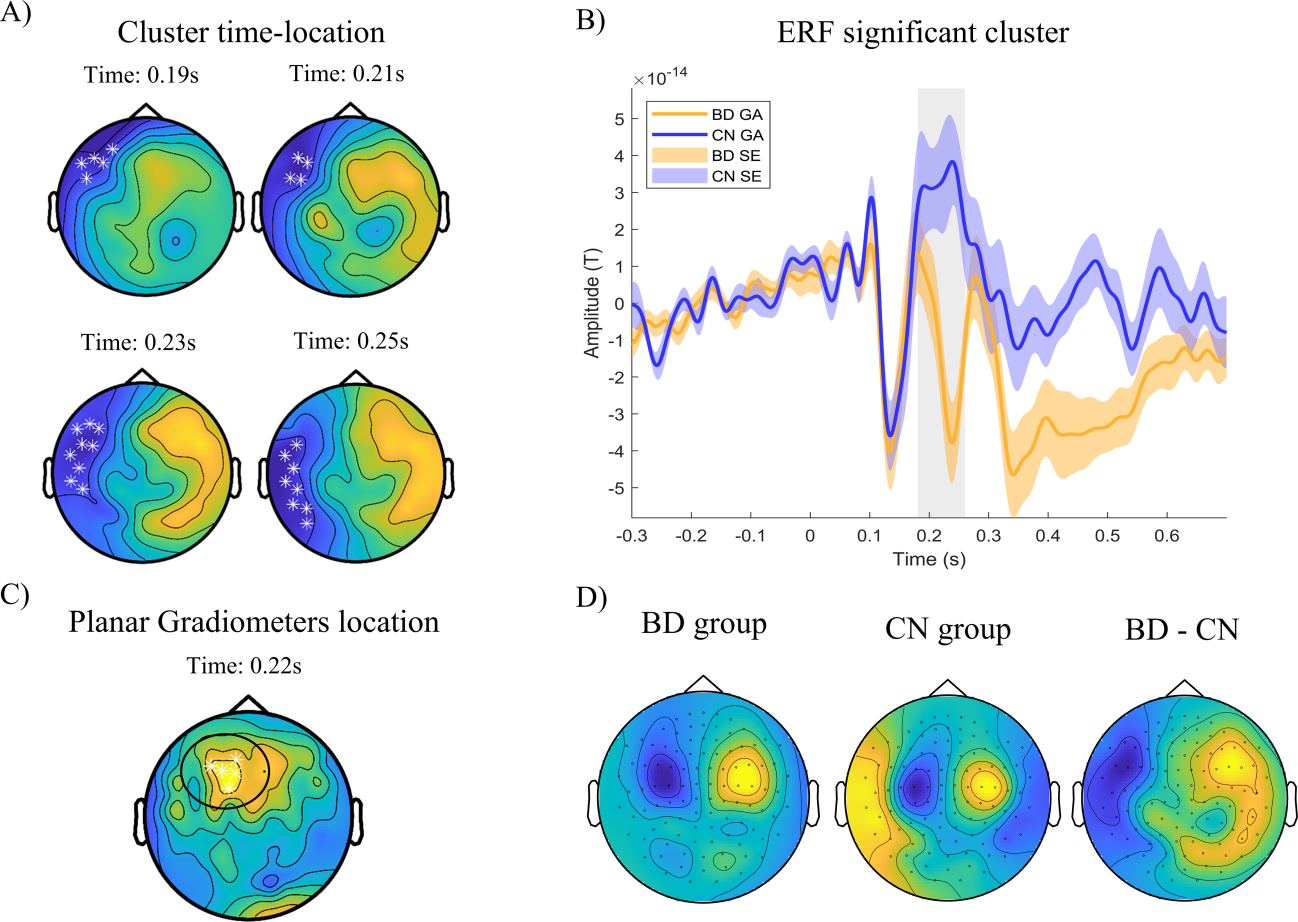
CBPT analysis: Event related fields results 50 – 300ms. Representation of significant cluster (M200) after CBPT analysis. **(A)** Cluster location and evolution through latencies. White asterisks represent sensors with significant differences between groups within the cluster (BD – CN; p 0.05). Blue colors represent negative statistical parameter F (BD > CN) and negative magnetic fields (inflow). Yellow colors represent positive statistical parameter F (BD < CN) and positive magnetic field (outflow). **(B)** ERF representation of cluster averaged activity across time. Grey shadowed rectangle represents time with significant differences between groups. BD group showed greater amplitude (distant from 0) in the latencies from 180 to 260ms post-stimulus. **(C)** Results of CBPT analysis using planar gradiometers. Significant activity locations appear centered across left and middle prefrontal sensors. **(D)** Representation of magnetometers amplitudes averaged across significant time window, for each group and their subtraction (BD - CN). GA, Grand Average; SE, Standard Error.

**Figure 3:**
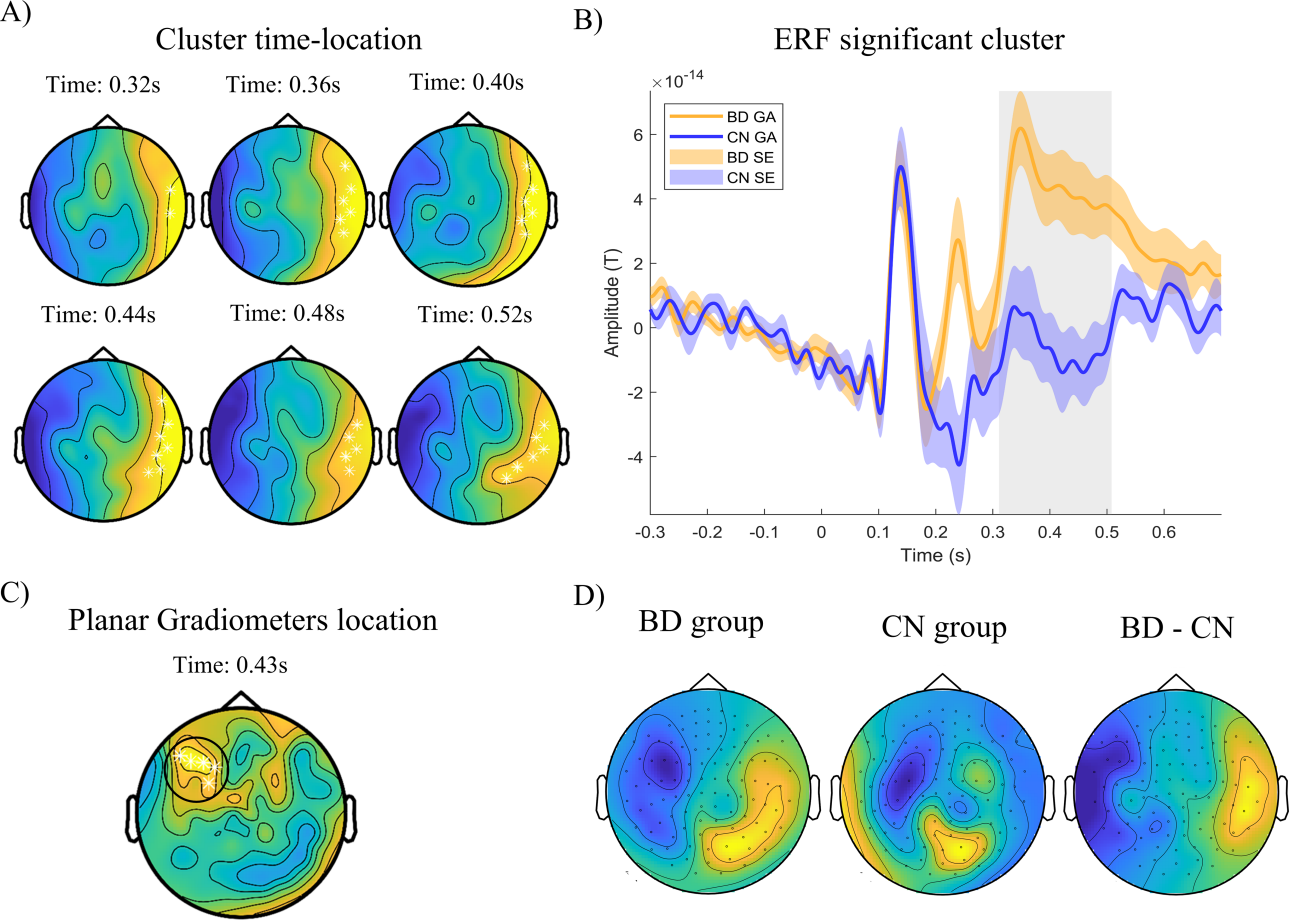
CBPT analysis: Event related fields results 250 – 600m. Representation of significant cluster (M300) after CBPT analysis. **(A)** Cluster location and evolution through latencies. White asterisks represent sensors with significant differences between groups within the cluster (BD – CN; *p* < 0.05). Blue colors represent negative statistical parameter F (BD < CN) and negative magnetic fields (inflow). Yellow colors represent positive statistical parameter F (BD > CN) and positive magnetic field (outflow). **(B)** ERF representation of cluster averaged activity across time. Grey shadowed rectangle represents time with significant differences between groups. BD group showed greater amplitude (distant from 0) in the latencies from 310 to 510ms post-stimulus. **(C)** Results of CBPT analysis using planar gradiometers. Significant activity locations appear centered across left prefrontal and parietal sensors. **(D)** Representation of magnetometers amplitudes averaged across significant time window, for each group and their subtraction (BD - CN). GA, Grand Average; SE, Standard Error.

Regarding the ERF analysis, we conducted a cluster-based permutation test (CBPT) using an ANCOVA model with age and sex as covariates to identify clusters of group differences across sensor locations and time points. The analysis focused on two-time intervals corresponding to the M200 (50–300 ms) and M300 (250–600 ms) components. Within the M200 interval, we observed a significant cluster differentiating between the BD and CN groups (permuted *p* = 0.011). The strongest effects emerged between 180 and 260 ms (**Figure 2b**), spanning from left prefrontal sensors at earlier latencies to left frontotemporal sensors at later latencies, corresponding to the negative part of the magnetic dipole (18 sensors). Since the magnetic dipole measured with magnetometer sensors must be located at the midpoint between negative and positive field patterns, the origin of the activity is likely situated in the left medial prefrontal area, potentially involving subcortical structures given the distance between the magnetic poles (see **Figure 2a**). To confirm the precise sensor locations of these differences, we performed the same analysis with combined planar gradiometer sensors, which primarily capture local activity from dipoles located directly beneath the sensor. This analysis confirmed that the strongest differences were located in left and medial prefrontal sensor positions, with the BD group showing higher amplitudes (see **Figures 2c, d**). Within the P300 interval (250–600ms), we found a significant cluster of differences between BD and CN groups (permuted *p* = .008). Statistical differences were strongest from 310 to 510ms (**Figure 3b**), with a positive field over right parietal sensors extending toward medial parietal regions (12 sensors) and a negative field over left frontotemporal regions (see **Figure 3a**). According to the dipole configuration, the origin of the activity was located in the left prefrontal area. The analysis with combined planar gradiometer sensors confirmed that the strongest differences were observed in left prefrontal sites, possibly involving dorsolateral prefrontal regions, with higher amplitude in the BD group (see **Figures 3c, d**). In order to analyze potential effects of sensation seeking differences on this association, we introduced sensation seeking as an additional covariable in the CBPT test. Both M200 and M300 cluster differences remained significant with no changes in the latency, nor in the sensors involved.

### 3.3 Post-hoc analysis: logistic regression model and moderation of biological sex.

To evaluate the predictive potential of the ERF activity identified in our analyses, we conducted a logistic regression to determine whether these neural markers could predict subsequent alcohol use habits. The model included ERF activity from both significant clusters (M200 and M300), controlling for age and sex. To assess the robustness and generalizability of the models, a leave-one-out cross-validation procedure was applied. Classification performance was evaluated in terms of accuracy, sensitivity, specificity, positive predictive value (PPV), and negative predictive value (NPV). Accuracy represents the overall proportion of correctly classified participants, whereas sensitivity and specificity indicate the proportions of participants correctly identified as future heavy and low drinkers, respectively. Confidence intervals (CIs) for accuracy were calculated using the Clopper and Pearson (1937) method. Logistic regression models based on ERF activity from the M200 and M300 clusters demonstrated adequate predictive performance for future alcohol use. For the M200 cluster, the model achieved an accuracy of 77.3% (95% CI [62.2, 88.5]), with a sensitivity of 80.0% and a specificity of 75.0%. The cross-validation model showed comparable performance (accuracy = 72.7%, 95% CI [57.2, 85.0]). For the M300 cluster, the original model reached an accuracy of 70.5% (95% CI [54.8, 83.2]), with a sensitivity of 70.0% and a specificity of 70.8%, while the cross-validation model yielded an accuracy of 68.2% (95% CI [52.4, 81.4]). These findings indicate that ERF activity, particularly within the M200 cluster, provides reliable predictive information regarding subsequent alcohol use. **Table 2** summarizes the classification performance for both clusters and models.

**Table 2:** Classification performance of logistic regression models predicting alcohol use based on ERF activity (M200 and M300 clusters).

Model	ACC (95% CI)	SEN	SPE	PPV	NPV
M200 (original)	77.3 (62.2–88.5)	80.0	75.0	72.7	81.8
M200 (cross-validation)	72.7 (57.2–85.0)	75.0	70.8	68.2	77.3
M300 (original)	70.5 (54.8–83.2)	70.0	70.8	66.7	73.9
M300 (cross-validation)	68.2 (52.4–81.4)	70.0	66.7	63.6	72.7

ACC, accuracy; SEN, sensitivity; SPE, specificity; PPV, positive predictive value; NPV, negative predictive value. Accuracy values are reported with their 95% confidence intervals (CI). M200 and M300 activity showed moderate accuracy in the prediction of future group of consumption.

In order to explore potential influences of biological sex on the association between ERF activity and future alcohol consumption, we conducted two moderation analyses (one for each significant cluster). We used averaged ERF activity of each significant cluster (time and sensors) as the predictor and future alcohol use (SAUs) as the dependent variable, with sex as the moderator and age as a covariate. For the M200 cluster, the overall model was significant, [F(4, 39) = 4.22, p = .006] explaining approximately 30% of the variance in alcohol use (R² = .30). ERF activity significantly predicted alcohol use (b = –1.53, SE = 0.39, t = –3.91, p <.001; CI 95% [-2.31, -0.73]). These findings suggest that individuals showing stronger M200 responses (i.e., more negative amplitudes) are more likely to engage in higher alcohol use over time. The interaction between ERF activity and sex was not statistically significant, and its confidence interval included zero. Thus, based on the current sample, it cannot be concluded that the predictive association between M200 activity and alcohol use differs between males and females (b = –0.50, SE = 0.78, t = –0.64, p = .52; CI 95% [-2.07, 1.08]). For the M300 cluster, the overall model approached significance, F(4, 39) = 2.52, p = .057, accounting for approximately 21% of the variance in alcohol use (R² = .21). ERF activity significantly predicted alcohol use (b = 1.45, SE = 0.45, t = 2.85, p = .007, 95% CI [0.37, 2.21]), indicating that stronger M300 responses were associated with greater alcohol consumption at follow-up. The interaction between ERF activity and sex was not statistically significant (b = 1.59, SE = 1.04, t = 1.52, p = .14, 95% CI [-1.48, 2.07]), and its confidence interval included zero. Therefore, although increased M300 activity prospectively predicted alcohol use, the available evidence does not allow us to determine whether this association differs between males and females.

## 4 Discussion

This longitudinal study examined whether pre-consumption neurophysiological markers, obtained with MEG during a Go/No-Go task, predict vulnerability to adolescent binge drinking (BD) years later. Overall, differences in inhibitory control ERFs prior to drinking onset appear to manifest in neurophysiological components, especially the M200 and M300, rather than in behavioral performance. Adolescents which reported higher alcohol consumption two years later had higher ERFs amplitudes compared to those with lower or absence alcohol consumption.

At the behavioral level, no group differences were observed in No-Go accuracy, indicating that inhibitory performance was preserved at baseline in both BD and CN adolescents. This finding is consistent with prior longitudinal evidence showing that pre-consumption alterations in inhibitory control are not always detectable behaviorally (32). Similarly, studies in adolescents with established BD have also reported intact task accuracy despite neural alterations (24, 25, 45). Thus, our results suggest that behavioral measures alone may not capture early vulnerability to BD, and that electrophysiological indices provide greater sensitivity for detecting subtle inhibitory control differences preceding alcohol use.

Regarding electrophysiological differences, significant group effects were observed for both the M200 and M300 components, contrary to the expectation that early inhibitory alterations would manifest as reduced amplitudes. The M200 electrophysiological event-related component, typically linked to conflict monitoring and the initiation of inhibition (18, 19), showed higher amplitudes in BD adolescents, localized in left medial and frontotemporal regions. Similarly, the M300, associated with attentional allocation and cognitive control (20), also revealed greater amplitudes in BD participants, with activity pointing to left dorsolateral prefrontal areas. Both patterns of neural activation emerged as moderate predictors of subsequent consumption habits, successfully classifying above 75% of participants into future low or heavy drinkers. These findings align with prior ERP studies reporting hyperactivation and enhanced No-Go P300 amplitudes in young BD users (25, 26), which have been interpreted as compensatory recruitment to sustain performance despite underlying inefficiencies. They also converge with developmental MEG evidence showing that adolescents, compared to adults, recruit prefrontal networks more extensively during inhibition, reflecting the ongoing specialization of the inhibitory system (16). Within this framework, the increased M200 and M300 amplitudes observed in BD adolescents may represent an accentuation of a maturational pattern in which greater prefrontal engagement is needed to maintain performance. Interestingly, such maturational pattern associated with ERF activation, did not seem to be influenced by biological sex, suggesting that these neural predictors, based on inhibitory control activity, are consistent across males and females. However, further investigations should confirm this lack of association between ERP biomarkers and biological sex in larger samples enhancing statistical power.

The neural location of these components provides further support for this interpretation. The M200 has been consistently localized to medial and lateral prefrontal regions, particularly the anterior cingulate cortex (ACC), dorsolateral prefrontal cortex (DLPFC), and the right inferior frontal gyrus (IFG), which support conflict monitoring and inhibitory control (14, 19, 46). Developmental evidence suggests a broader involvement of left prefrontal regions during adolescence, consistent with the topography of our results (16, 33). The M300, in turn, reflects later stages of control and has distributed generators: the P3a, typically frontocentral, involves the DLPFC and ACC and supports attentional orienting, whereas the P3b, with parietal and hippocampal contributions, is linked to stimulus evaluation and working memory updating (20). Taken together, the enhanced M200 and M300 amplitudes in left prefrontal areas observed in BD adolescents suggest the recruitment of additional neural resources in the inhibitory networks at a developmental stage where these systems may be still maturing.

At the same time, our findings resonate with pre-consumption MEG evidence from an independent cohort, where altered connectivity in left prefrontal inhibitory networks was also linked to later BD onset (33). The convergence across samples suggests that atypical prefrontal recruitment may constitute an early marker of risk. Conversely, evidence from youth with a family history of AUD consistently points to a different electrophysiological profile, reduced P300 amplitudes and delayed N200 latencies (35–37), that appears to reflect inherited inefficiency of the inhibitory system. Taken together, this body of work raises the hypothesis of two distinct vulnerability pathways: 1) a maturational profile in the general adolescent population, characterized by compensatory hyperactivation of prefrontal regions. According to this hypothesis, adolescent neurodevelopment follows heterogeneous pathways with divergent maturational trajectories. Individuals showing delayed or inefficient development of prefrontal executive-control networks may be more vulnerable to difficulties in behavioral regulation, which in turn may increase susceptibility to risky behaviors, such as alcohol misuse. At the electrophysiological level, these profiles would likely display compensatory recruitment of additional neural resources to meet environmental demands and sustain adequate functioning. And 2) a familial–genetic pathway, characterized by reduced neural efficiency and attenuated electrophysiological responses, which may compromise inhibitory control at a relatively early stage. This distinction underscores heterogeneity in trajectories of risk for BD and substance use disorders during adolescence, emphasizing the need for further longitudinal studies to clarify developmental mechanisms and long-term outcomes.

BD and CN groups did not differ in trait impulsivity (BIS−11), or self−reported everyday executive problems (BRIEF−SR), but BD adolescents reported significantly higher sensation seeking (SSS−V). This pattern fits dual−systems accounts in which the socioemotional system matures earlier than prefrontal control, elevating sensation seeking during mid−adolescence (11, 12). Current results of higher sensation seeking in BD adolescents align with prior evidence showing this trait as a robust predictor of risky drinking. Recent studies with MEG functional connectivity (47, 48) found that high sensation seeking was linked to resting-state hyperconnectivity and poorer regulation before alcohol initiation. On the other hand, longitudinal studies have shown that sensation seeking predicts escalation in alcohol use (49, 50). Consistent with this, Castelli et al. (2022) (51) demonstrated in pre-clinical models that binge-like alcohol exposure during adolescence induces lasting emotional dysregulation, blunted stress reactivity, and maladaptive behavioral responses. Together, these findings suggest that sensation seeking functions both as a behavioral driver and as a marker of neural vulnerability for adolescent binge drinking. Given the observed group differences in sensation seeking in our sample, we examined whether this variable influenced the electrophysiological results by including it as a covariate in the ERF models. Importantly, the significant group effects on M200 and M300 amplitudes remained, indicating that these neural differences cannot be attributed solely to variability in sensation seeking. This pattern suggests that, although sensation seeking may increase exposure to risk contexts, the electrophysiological alterations are more likely to reflect underlying differences in inhibitory control that heighten vulnerability to engage in risky drinking. Thus, this evidence highlights how behavioral and emotional dysregulation can emerge during key maturational periods, such as pubertal transition, and may subsequently be exacerbated by harmful patterns of alcohol use, including binge drinking.

A major strength of this study is the use of MEG, which provides high temporal resolution together with improved spatial reliability compared to traditional ERP methods, allowing a precise characterization of the dynamics of inhibitory control. The longitudinal design adds further value by enabling the identification of neural alterations that precede BD onset, while the application of robust statistical approaches, including cluster-based permutation analyses, strengthens the validity of the findings. Nonetheless, some limitations must be acknowledged. The sample size was relatively modest, which may limit statistical power and generalizability, and future studies with larger cohorts needed to confirm these results. In addition, while sensor-level analyses revealed reliable group differences, further work using source reconstruction is required to confirm the specific cortical generators involved in the observed electrophysiological alterations.

In conclusion, our findings indicate that adolescents who later engage in BD already show electrophysiological differences during inhibitory control processes, characterized by enhanced M200 and M300 amplitudes localized in left prefrontal regions, despite the absence of behavioral impairments. This pattern supports the view that atypical recruitment of inhibitory networks precedes the onset of alcohol use and may represent a maturational vulnerability profile that requires compensatory engagement of prefrontal resources. Although BD adolescents also reported higher sensation seeking, the electrophysiological differences remained after controlling for this factor, underscoring that neural alterations are not reducible to personality traits alone. Taking together, these results highlight prefrontal hyperactivation as a potential early biomarker of risk for BD, reinforcing the need for longitudinal approaches to disentangle developmental vulnerabilities from the effects of alcohol exposure.

## Data Availability

The raw data supporting the conclusions of this article will be made available by the authors, without undue reservation.
